# Identification of Important and Relevant Functioning‐Based Outcomes for Persons With an Oral Health Condition From the Patient's Perspective

**DOI:** 10.1111/joor.70078

**Published:** 2025-10-15

**Authors:** C. Lenherr, M. Schimmel, G. Stucki, M. Selb

**Affiliations:** ^1^ Faculty of Health Sciences and Medicine University of Lucerne Lucerne Switzerland; ^2^ Department of Reconstructive Dentistry and Gerodontology, School of Dental Medicine University of Bern Bern Switzerland; ^3^ ICF Research Branch Nottwil Switzerland; ^4^ Swiss Paraplegic Research Nottwil Switzerland

**Keywords:** disability and health, functioning, international classification of functioning, oral health, patient‐centered care, qualitative research

## Abstract

**Background:**

The aim of this study was to identify the most important and relevant aspects of functioning related to oral health from the patient's perspective.

**Materials and Methods:**

In this multicenter cross‐sectional qualitative study, focus groups and semi‐structured interviews were conducted. Adults age 18 years or older with an oral health condition, such as caries, periodontal disease, cancer of the oral cavity or lips, were included. The content of the focus groups and interviews was recorded, transcribed, and analysed using thematic analysis, that is, patterns in the data were identified and broken down into meaningful concepts, which in turn were linked to the most precise categories of the International Classification of Functioning, Disability and Health (ICF). Focus groups and interviews were analysed until saturation was reached.

**Results:**

Statements of 36 participants were included in the analysis. Saturation was reached after analysing five focus groups and 3 interviews, 1213 meaningful concepts were identified and subsequently linked to 150 ICF categories (51 in the Body Functions component, 39 in Activities and Participation, 16 Body Structures, 44 in Environmental Factors) and 102 Personal Factors. Thirty‐four concepts were not covered by the ICF and 49 were non‐definable concepts. The results of one focus group comprising of young health professionals are displayed separately.

**Conclusion:**

Functioning‐based outcomes for persons with an oral health condition were identified in every component of the ICF, indicating that oral health conditions affect different aspects of an individual's everyday life.

## Graphical abstract

Overview of the qualitative study “Identification of Important and Relevant Functioning‐Based Outcomes for Persons with an Oral Health Condition from the Patient's Perspective.” The study involved 36 adults with various oral health conditions and used focus groups and interviews analyzed thematically and linked to the ICF framework. Results covered all ICF components, demonstrating that oral health conditions affect multiple aspects of individuals' daily functioning and participation.

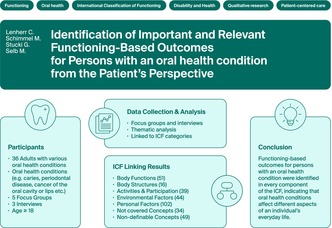

## 1 Introduction

According to the World Health Organization (WHO), oral health is key for general health, well‐being and quality of life, encompassing various health conditions such as caries, periodontal disease and oral cancer. Oral health conditions are ranked among the most prevalent conditions worldwide [1]. Among those most affected are older persons, people with disabilities or long‐term illness and socially‐excluded populations [2, 3]. Oral health conditions affect approximately 3.5 billion people worldwide; of these, 2.3 billion have untreated caries, 796 million severe periodontitis, 267 million have tooth loss. The prevalence of oral health issues is rising, particularly in low‐ and middle‐income nations [1, 4].

Furthermore, there are correlations between oral and specific medical conditions, for example, periodontal disease and diabetes mellitus or tooth loss and cardiovascular disease [5]. Nevertheless, oral health care is often underrecognized and undervalued in public health systems [1]. This has led to tremendous unmet needs, especially in the geriatric population and other vulnerable groups [1, 2].

Recognising this need, WHO presented a global plan on oral health at the 2022 World Health, aiming to ensure the overall population health by addressing oral health issues [6]. WHO defines oral health as multifaceted, moving away from considering oral health and care from solely the absence of disease to a more integrated biopsychosocial perspective. This definition is also captured in the World Dental Federation definition of oral health [6, 7, 8]. Functioning is the term that WHO uses to reflect this biopsychosocial perspective of health, and the internationally recognised framework for operationalising functioning is WHO's International Classification of Functioning, Disability and Health (ICF) [9].

Functioning is the third indicator of health alongside mortality and morbidity, and it is a key indicator for rehabilitation, one of the five health strategies [10, 11, 12, 13]. When looking at rehabilitation in the context of the ICF, rehabilitation aims to optimise functioning, particularly a person's intrinsic health and daily activities. This can be achieved through quality medical (including dental) care or by minimising constraints in social and physical contexts [12, 14]. For example, oral rehabilitation addresses the health of the mouth and face, by restoring the ability to eat, speak, and communicate, while also considering biological, psychological and social aspects. As current diagnostic and assessment tools in oral health do not capture these concepts of functioning, there is a need for more comprehensive standardised tools and measures [7, 8, 15].

**FIGURE 1:**
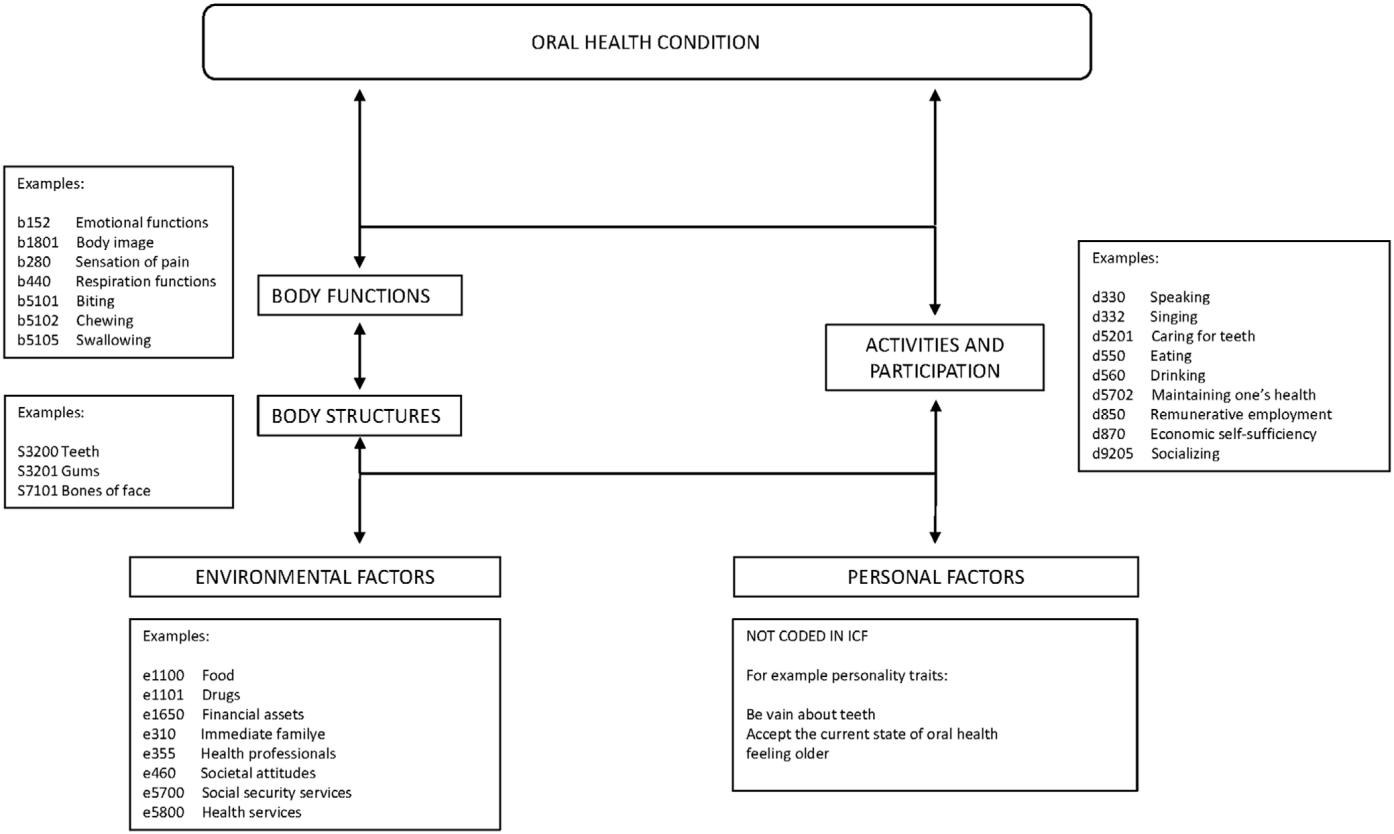
Biopsychosocial framework of the International Classification of Functioning, Disability and Health with examples from an oral health perspective.

The ICF can support the development of such tools and measures by providing an internationally accepted reference system that can be used to gather information about functioning and health that is uniform and globally comparable. The ICF can be used to describe not only a person's functioning and their ability to participate in life situations, such as work or community involvement, but also the environmental and personal factors that influence everyday life (see Figure 1) [9].

The present study aimed to identify the most important and relevant aspects of functioning related to oral health from the patient's perspective. It is one of the essential preparatory steps in the established scientific process for developing an ICF Core Set—a concise list of functioning outcomes represented as ICF categories [16]. There are four main types of preparatory studies, each capturing a distinct perspective: patient, researcher, expert, and clinical setting. This study focuses on the patient's perspective and serves as the first step in developing standards and tools for the comprehensive, functioning‐based assessment of individuals with oral health conditions.

To this end, the ICF was used as a conceptual framework to systematically structure and compare patients' perspectives on functioning and health. By aligning individual experiences with internationally defined categories, this approach contributes to the development of a globally applicable ICF Core Set for oral health while ensuring that the patient voice remains central.

## 2 Methods

### 2.1 Study Design

We conducted a multicenter qualitative study using focus groups and semi‐structured interviews to explore the lived experiences and functioning of individuals with oral health conditions from their own perspective. This study takes a participant‐centered approach, focusing on subjective experiences.

Focus groups were chosen to facilitate shared reflection and dynamic discussion among participants, enabling the emergence of collective insights. Individual interviews were offered to those who preferred to share their experiences privately or could not attend a group discussion.

The study was conducted in the German‐speaking part of Switzerland.

Ethics approval was granted by both the “Kantonale Ethikkommission Bern” and the “Ethikkommission Nordwest‐ und Zentralschweiz” (Project‐ID 2023‐00405).

The presentation of the methods and reporting of the results are aligned with the Consolidated Criteria for Reporting Qualitative Research (COREQ) reporting guidelines [17].

### 2.2 Researcher Experience/Background

The main study investigator is a dentist with clinical experience and is currently pursuing a PhD in Health Sciences. She conducted all focus groups, semi‐structured interviews, and the primary coding and analysis. She received formal training in ICF linking and qualitative research methods through the official online ICF Workshop offered by the ICF Research Branch.

The second investigator, who is a senior researcher with extensive experience in ICF‐based research and is the Coordinator of the ICF Research Branch, contributed to study oversight and methodological rigour. She attended the first focus group and the first interview to provide feedback on data collection procedures and independently coded a non‐random sample comprising 15% of the transcribed text. In addition, she conducted the full ICF linking process for all coded data, and consensus was reached in discussion with the main study investigator.

### 2.3 Participants

Participants were adults (aged 18 and older) living with an oral health condition, such as but not limited to, the four main oral health conditions—caries, periodontal disease, cancer of the oral cavity or lips [1]. Inclusion criteria required the ability to speak and understand German to ensure participants could comprehend the study's purpose, complete the informed consent form and a designated questionnaire. Individuals under the age of 18 or with insufficient German language skills were excluded.

The study was conducted in the cantos of Bern and Lucerne, where Swiss German—a predominantly spoken dialect of Standard German without an official written form—is commonly used in everyday communication. For written materials, Standard German is used; accordingly, both the consent form and the questionnaire were provided in Standard German. If all participants agreed, focus group discussions were held in Swiss German to ensure participants—especially older individuals—could express themselves comfortably in their native dialect.

No upper age limit was set for a broad age range and a diverse participant sample. Participants were informed that the main study investigator is a practicing dentist and PhD student at the University of Lucerne, and that the second investigator is an experienced researcher. None of the final participants had a personal or professional relationship with either investigator.

Participants who attended a focus group or semi‐structured interview were given a 20 Swiss Franc Gift card for a Swiss supermarket chain.

#### 2.3.1 Note on Excluded Group

One focus group initially included young health professionals with prior affiliation to the faculty of medicine. Although they formally met the inclusion criteria, their contributions reflected a professional rather than a patient perspective. As this did not align with the study's primary aim of exploring patient experiences, this group was excluded from the main analysis. However, their data were retained and analysed separately and are reported independently to ensure transparency.

### 2.4 Recruitment

Participant recruitment was primarily conducted by the main study investigator in two clinical settings: a private dental practice in Muri and the Dental Clinic of the School of Dental Medicine at the University of Bern (ZMK Bern). In addition, several other dental practices in the regions of Bern and Lucerne were contacted and invited to support the recruitment process.

Dental practices that agreed to participate received a study announcement poster, an informational fact sheet outlining their responsibilities, and folders containing the participant information sheet, informed consent form, and a printed questionnaire. All documents were also made available via QR code. Dentists or clinical staff were instructed by the project team on the study procedures and how to identify and approach eligible patients.

Patients who met the inclusion criteria were informed about the study and invited to participate. Oral health conditions were confirmed either by reviewing patients' medical records or by the treating dentist; participants were not asked to self‐report their diagnoses.

Participants were recruited from the following three settings:

- Private dental practice in Muri: A rural practice selected to represent non‐urban patients.
- Private dental practice in Bern: A city‐center practice reflecting urban care settings.
- Dental Clinic of the School of Dental Medicine, University of Bern (ZMK Bern): A university‐based clinic representing a socioeconomically and clinically diverse population, including patients treated by dental students under supervision.

The study was also announced online via the Swiss Society of Periodontology website (www.parodontologie.ch) and on social media platforms (Instagram via the second author's personal account and Facebook via ZMK Bern). However, no participants were recruited through these online channels.

### 2.5 Sampling

The study protocol aimed to use purposeful sampling, with the goal of capturing a wide range of experiences related to oral health conditions. This strategy was chosen to allow common themes to emerge from variation among participants [18]. However, the final composition of the sample was influenced by the availability and willingness of individuals to participate, resulting in a convenience sample.

To preserve the intent of maximal variation sampling, we selected three distinct dental care settings for recruitment: (1) a rural private practice, (2) an urban private practice, and (3) a university dental clinic. This approach was designed to capture perspectives across different care settings, socio‐economic backgrounds, and clinical profiles, including individuals with complex oral health needs.

We applied only minimal exclusion criteria (age under 18 and insufficient German language skills), enabling broad participation. This inclusive strategy increased the likelihood of diversity in terms of age, gender, socio‐economic status, oral health conditions, and types of care received. While practical constraints shaped the final sample, the design and recruitment strategy remained consistent with the principles of maximal variation sampling.

### 2.6 Data Collection

Data collection consisted of focus groups and semi‐structured interviews, conducted by the main study investigator. Participants were asked to bring their signed informed consent form and completed questionnaire to the scheduled session. Both focus groups and interviews were audio‐recorded, and field notes were taken to capture non‐verbal communication and contextual information.

The secondary investigator was present during the first focus group and the first interview to provide structured feedback on data collection procedures. No repeat interviews were conducted. Transcripts and study findings were not returned to participants for comment or verification.

#### 2.6.1 Questionnaire

The questionnaire collected sociodemographic and general health‐related information. It was developed by the research team based on instruments used in previous qualitative studies in the context of ICF Core Set [19] and was adapted to reflect characteristics relevant to individuals with oral health conditions.

While not formally validated, the questionnaire was designed to be concise and relevant, providing descriptive background information to support the interpretation of qualitative findings. Participants could complete it on paper or electronically via RedCap, a secure web‐based data management platform. Paper responses were later entered into RedCap.

The questionnaire is available in the Supporting Information.

#### 2.6.2 Focus Groups

Focus group discussions were conducted in neutral, non‐clinical locations, for example in a meeting room at the Dental Clinic of the School of Dental Medicine in Bern (ZMK Bern) or at the municipal office in Muri. All focus groups were led by the main study investigator. The second investigator was present for the first focus group and the first interview that was conducted to provide feedback on facilitation techniques.

Each discussion lasted approximately 1 h and was audio‐recorded with the consent of participants. Field notes were taken by the main study investigator to capture non‐verbal communication (e.g., gestures) and contextual impressions.

The discussions were guided by a structured PowerPoint presentation, using a set of eight open‐ended questions reflecting all ICF components [9]. The following questions were used to initiate and structure the discussions:

When you think about your oral health/dental problem in your everyday life…

1. In which area of your face or mouth is your problem located and are there other parts of your body associated with it?
2. What physical and mental problems are you experiencing?
3. How do you feel when you look at yourself in the mirror?
4. What do you find difficult in your daily activities and daily routine?
5. What do you find difficult at work, at school, in social interactions or in the community?
6. Who or what in your environment or living situation do you find supportive?
7. Who or what in your environment or living situation restricts you?
8. What personal characteristics play a role in how you deal with this problem?

The order in which participants spoke varied and an open discussion was encouraged throughout. The main study investigator ensured all participants had the opportunity to contribute.

#### 2.6.3 Semi‐Structured Interviews

Semi‐structured interviews were conducted with participants who preferred an individual setting or were unable to attend a focus group. All interviews were conducted by the main study investigator and lasted approximately 30 min.

Each interview was audio‐recorded with the participant's consent and guided by the same set of open‐ended questions used in the focus groups. This ensured consistency in data collection across both formats while allowing for individual elaboration and depth.

### 2.7 Data Analysis

All audio recordings from focus groups and interviews were transcribed verbatim by the main study investigator. Transcriptions were completed manually without the use of artificial intelligence tools. The transcripts were produced in Standard German, while preserving sentence structures and terminology characteristic of Swiss German as used by participants.

Transcription was carried out concurrently with data collection. Transcripts were not returned to participants for comment or correction.

#### 2.7.1 Coding and Linking to the ICF

We applied thematic analysis [20, 21, 22] to identify and code patterns of meaning within the data. This approach allowed us to capture concepts relevant to participants' lived experiences of oral health and functioning. Thematic analysis began with familiarisation with the transcripts and the extraction of meaningful concepts—defined as discrete segments of text representing a single idea. These were identified inductively from the transcribed focus groups and interviews.

To avoid overweighting individual perspectives, concepts that were repeated by the same participant were coded only once, even if mentioned multiple times. Conversely, if similar concepts were expressed by different participants, each instance was coded separately to preserve the uniqueness of individual perspectives and indicate collective relevance. Field notes were consulted to help interpret statements that were unclear or influenced by non‐verbal cues, such as tone of voice or gestures.

At this point, we deliberately diverged from the classic thematic analysis process, which would typically involve developing higher‐order themes. Instead, we used the ICF framework to categorise and structure the data by linking each meaningful concept to the most specific applicable ICF category or ICF categories [23]. For an example, see Table 1, where bold text highlights a discrete segment of text representing a single idea, which served as the basis for identifying the corresponding meaningful concept. In line with an inclusive and transparent approach, any ICF category mentioned at least once in a focus group or interview was retained in the aggregated outcomes list for that session, regardless of frequency.

**TABLE 1:** Qualitative data analysis and linking—Example from focus group 1 translated in English.

Extract from transcript	Meaningful concept	ICF code
“… especially if you have fewer *teeth*, you can't **bite** everything, I couldn't *bite* into an apple for a long time, I always had to cut it up, and **over time I changed my diet so that I ate things that I could *bite* into. I suddenly preferred a soft apricot to an apple**, which I could at least *bite* and **chew**, and now I have teeth everywhere again, now I can bite everything again, I'm very happy about that”	Bite	b5101 biting
	Change diet	d5701 managing diet and fitness
	Chew	b5102 chewing

*Source:* WHO, International Classification of Functioning, Disability and Health (ICF), 2001.

All coded concepts were independently linked to the ICF by both investigators. Discrepancies were discussed and resolved by consensus, in accordance with the linking rules and the context of the respective concepts. The linking was performed manually, without the use of automated software.

All analyses were conducted using MAXQDA Analytics Pro, with no themes generated or artificial intelligence tools. This ensured that the coding and linking process remained entirely manual and interpretive.

#### 2.7.2 Saturation of Data

Focus groups and individual interviews were analysed (i.e., coding and linking were completed) until saturation was considered reached, meaning no substantial new information emerged [24, 25]. Transcripts were analysed in chronological order of data collection. The transcripts were reviewed progressively, and saturation was calculated retrospectively after all data had been collected. For each newly analysed focus group or interview, the list of identified ICF categories was compared with the cumulative list from all previously analysed transcripts.

Saturation was quantified at each step using the following formula:

$$\begin{array}{c} \%\mathrm{new}\;\text{categories}=\left(\text{number of}\;\mathrm{new}\;\mathrm{ICF}\;\text{categories}/\right.\\ \;\left.\text{total}\;\mathrm{ICF}\;\text{categories identified}\;\mathrm{so}\;\mathrm{far}\right)\times 100 \end{array}$$

This step‐by‐step comparison was conducted across the nine transcripts to track newly emerging ICF categories at each stage. Consistent with prior ICF Core Set studies, saturation was considered reached when no more than approximately 5% new categories emerged between consecutive transcripts [26, 27].

#### 2.7.3 Data Quality

Regular spot checks of questionnaire data entries were conducted, and any missing data was promptly clarified with participants. To assess reliability in the qualitative coding process, the second investigator independently coded a non‐randomised sample comprising 15% of the transcribed text from each focus group and interview. These passages were then jointly reviewed, and consensus was reached through discussion. The remaining transcripts were coded independently by the main study investigator.

To ensure quality in the ICF linking process, both investigators independently linked all coded concepts to the ICF. The degree of agreement was calculated using the following formula:

$$\begin{array}{c} \text{Agreement}\;\left(\%\right)=\left(\text{Number of concepts with identical}\right)\\ \;\mathrm{ICF}\;\text{codes assigned}\;\mathrm{by}\;\text{both investigators}/\\ \left.\;\text{Total number of concepts reviewed}\right)\times 100 \end{array}$$

This approach allowed us to assess consistency in the application of the ICF linking rules and minimise potential bias in category assignment.

## 3 Results

### 3.1 Participants

Six focus groups and three interviews were conducted from June 2023 to July 2023, involving 36 participants. The personal background information of the participants, that was collected though the questionnaire, is shown in Table 2.

**TABLE 2:** Characteristics of participants.

Characteristics		
Age, years, mean (SD)	63.72	18.8
	***n***	**%**
Gender		
Male	22	
Female	14	
Civil status		
Never married	9	25
Married	17	47.2
Divorced	7	7
Widowed	2	2
Engaged	1	1
Living status		
Alone	10	27.8
With another person	26	72.2
Work status		
Employed	11	30.6
Self‐employed	1	2.8
In education/Student	3	8.3
Housewife/Househusband	1	2.8
Retired	18	50
Unemployed	1	2.8
Unable to work	1	2.8
Level of education		
Primary education	2	5.6
Secondary education	1	2.8
Apprenticeship	17	47.2
Swiss University entrance qualification	1	2.8
University/University of applied sciences	11	30.6
Doctorate	1	2.8
Others: individual courses, advanced specialised training, business school	3	8.3
Monthly income		
Up to 5000 Swiss Francs	15	41.7
5000–10 000 Swiss Francs	17	47.2
10 000–15 000 Swiss Francs	2	5.6
More than 20 000 Swiss Francs	2	5.6
Smoker status		
Non‐smoker	15	41.7
Former smoker	9	25
Smoker	12	33.3
General diseases–multiple choice		
Arthritis	2	2.7
Arthrosis	9	12.3
Chronic respiratory disease	2	2.7
Diabetes	5	6.9
Cardiovascular disease	8	11.0
No general diseases	9	12.3
Cancer	4	5.5
Osteoporosis	2	2.7
Rheumatism	6	8.2
Back pain	9	12.3
Stress or depression	7	9.6
Other: Hashimoto, epilepsy, iron deficiency, hyperthyroidism, high blood pressure, multiple sclerosis, gastric bypass, migraine, chronic headache, Morbus Sjögren, gastroesophageal reflux disease	10	13.7
Problems with–multiple choice		
Aesthetic of the face	2	5
Breathing	1	2.5
Biting	9	22.5
Eating with others	2	5
Yawning	2	5
Taste	4	10
Chewing	7	17.5
Kissing	1	2.5
Swallowing	1	2.5
Singing	1	2.5
Salivation	3	7.5
Speaking	1	2.5
Tactile sensation in oral cavity	1	2.5
Dry mouth	5	12.5
Pain		
No pain	27	75
Pain comes and goes	9	25
Personal oral hygiene		
Three times a day and more frequently	9	25
Normally two times a day	27	75
Dental prothesis		
Fixed prothesis	22	66.7
Removable prothesis	11	33.3

The focus groups and interviews are listed below in the chronological order in which they were conducted:

1. Focus group with 7 participants

2. Interview with 2 participants

3. Focus group with 5 participants

4. Focus group with 4 participants

5. Interview with 2 participants

6. Interview with 1 participant

7. Focus group with 4 participants

8. Focus group with 7 participants

9. Focus group with 4 participants (young health professionals group)

The number of patients who initially declined participation prior to referral to the study team is unknown, as initial recruitment was conducted by the participating dental clinics and practices. Only individuals who expressed interest and provided consent to participate were referred to the research team and received a participant number. Among those referred 11 participants did not end up participating in the study.

### 3.2 Data Analysis

#### 3.2.1 Data Saturation

To assess whether saturation was reached, the newly identified ICF categories from each transcript were compared to the cumulative list of previously identified categories. After analysing eight transcripts (five focus groups and three interviews), the final transcript in this main sequence yielded only 8 new categories out of a cumulative 142. This corresponds to a saturation rate of 5.63% (8/142 × 100), indicating that few new categories emerged and saturation was being approached.

In contrast, the last focus group—comprising only young health professionals with oral health conditions—yielded 22 new ICF categories not previously identified. This represents a saturation rate of 14.7% (22/150 × 100), suggesting that this subgroup contributed unique perspectives. Due to their distinct profile and the emergence of new content, their results are presented separately (see “Focus group with young health professionals”).

#### 3.2.2 Degree of Agreement

To assess consistency in ICF linking, both investigators independently linked all extracted meaningful concepts to ICF categories. Their results were then compared, and a percentage agreement was calculated by assigning a value of “1” when the final code(s) matched, and “0” when a discrepancy had to be resolved through discussion. Across all six focus groups and three interviews, the overall agreement rate was 64%. The degree of agreement improved as the analysis progressed. Participants did not provide any feedback on the finding.

### 3.3 Relevant Aspects of Functioning

In five focus groups and three individual interviews a total of 1213 meaningful concepts were extracted/coded and subsequently linked to a total of 150 ICF categories. The 150 categories comprise of 51 Body Functions, 39 Activities and Participation categories, 16 Body Structures, and 44 Environmental Factors. 102 categories were Personal Factors, 34 were not covered and 49 non‐definable concepts.

The ICF categories that were mentioned in all the focus groups and interviews were b5101 Biting, b5201 Caring for teeth, d5702 Maintaining one's health, e310 Immediate family, e5800 Health services, s320 Structure of mouth and s3200 Teeth. Table 3 represents the ICF categories sorted by the number of focus groups/interviews in which they were mentioned. Additionally, the table displays the absolute frequency of mentions across all focus groups and interviews.

**TABLE 3:** ICF codes and concepts.

ICF code	ICF concept	Frequency (absolute) across all focus groups and interviews	Number of focus groups and interviews in which the category was mentioned (*N* = 8)
b5101	Biting	16	8
d5201	Caring for teeth	72	8
d5702	Maintaining one's health	43	8
e310	Immediate family	29	8
e5800	Health services	88	8
s320	Structure of mouth	13	8
s3200	Teeth	46	8
b28010	Pain in head and neck	14	7
d550	Eating	22	7
e1158	Products and technology for personal use in daily living, other specified‐implant	18	7
e355	Health professionals	45	7
e5801	Health systems	36	7
b152	Emotional functions (G)	20	6
d240	Handling stress and other psychological demands	7	6
d5701	Managing diet and fitness	12	6
d740	Formal relationships	8	6
d870	Economic self‐sufficiency	27	6
d9205	Socialising	20	6
e1100	Food	10	6
e1158	Products and technology for personal use in daily living, other specified—interdental brush	10	6
e1158	Products and technology for personal use in daily living, other specified—bridge	12	6
e325	Acquaintances, peers, colleagues, neighbours and community members	10	6
s710	Structure of head and neck region	10	6
b1801	Body image	32	5
d349	Communication—producing, other specified and unspecified—laughing	11	5
e1101	Drugs	19	5
e1158	Products and technology for personal use in daily living, other specified—prothesis	18	5
e1158	Products and technology for personal use in daily living, other specified—electric toothbrush	6	5
e1158	Products and technology for personal use in daily living, other specified—toothbrush	7	5
e1650	Financial assets	9	5
s3201	Gums	8	5
b28018	Pain in body part, other specified_headache	7	4
b28018	Pain in body part, other specified_toothpain	11	4
b5102	Chewing	5	4
d560	Drinking	4	4
d850	Remunerative employment (G)	7	4
e1158	Products and technology for personal use in daily living, other specified—toothfloss	9	4
e320	Friends	7	4
e5700	Social security services	14	4
s7101	Bones of face	8	4
b1265	Optimism	7	3
b1266	Confidence	6	3
b265	Touch function	6	3
b280	Sensation of pain (G)	4	3
b28018	Pain in body part, other specified_mouthpain	4	3
b515	Digestive functions	3	3
d760	Family relationships	4	3
d7600	Parent–child relationships	3	3
d8451	Maintaining a job	6	3
e1158	Products and technology for personal use in daily living, other specified—crown	3	3
e1158	Products and technology for personal use in daily living, other specified—toothpick	3	3
e315	Extended family	5	3
s32008	Other specified teeth—root of tooth	4	3
s3203	Tongue	4	3
b1300	Energy level	3	2
b140	Attention functions	2	2
b4350	Immune response	3	2
b4552	Fatigability	2	2
b5105	Swallowing	2	2
b530	Weight maintenance functions	3	2
d230	Carrying out daily routine (G)	4	2
d330	Speaking	2	2
d332	Singing	4	2
d7400	Relating with persons in authority	3	2
d7701	Spousal relationships	2	2
e1158	Products and technology for personal use in daily living, other specified—denture	2	2
e1158	Products and technology for personal use in daily living, other specified—braces	2	2
e1158	Products and technology for personal use in daily living, other specified—mouthguard	2	2
e1158	Products and technology for personal use in daily living, other specified—Amalgam Filling	5	2
e1158	Products and technology for personal use in daily living, other specified—post crown	2	2
e330	People in positions of authority	2	2
e350	Domesticated animals	2	2
e410	Individual attitudes of immediate family members	2	2
e460	Societal attitudes	2	2
b1260	Extraversion	1	1
b1301	Motivation	1	1
b134	Sleep functions	1	1
b1470	Psychomotor control	1	1
b1564	Tactile perception	1	1
b210	Seeing functions	1	1
b2153	Functions of lachrymal glands	1	1
b250	Taste function	2	1
b260	Proprioceptive function	1	1
b2700	Sensitivity to temperature	2	1
b2701	Sensitivity to vibration	1	1
b2702	Sensitivity to pressure	1	1
b28016	Pain in joints	1	1
b28018	Pain in body part, other specified_pain in neck	3	1
b28018	Pain in body part, other specified_pain in ears	1	1
b28018	Pain in body part, other specified_facepain	1	1
b320	Articulation functions	2	1
b435	Immunological system functions	1	1
b440	Respiration functions	1	1
b4500	Functions of breathing through the mouth	2	1
b5103	Manipulation of food in the mouth	1	1
b5104	Salivation	1	1
b5151	Breakdown of food	1	1
b5152	Absorption of nutrients	2	1
b520	Assimilation functions	1	1
b5350	Sensation of nausea	1	1
b5351	Feeling bloated	1	1
b7101	Mobility of several joints	1	1
b7300	Power of isolated muscles and muscle groups	1	1
b735	Muscle tone functions	1	1
b7651	Tremor	2	1
b7801	Sensation of muscle spasm	1	1
d175	Solving problems	1	1
d2301	Managing daily routine	1	1
d3150	Communicating with—receiving—body gestures	4	1
d350	Conversation	2	1
d440	Fine hand use	1	1
d470	Using transportation	2	1
d5208	Caring for body parts, other specified_putting on make‐up	1	1
d640	Doing housework	1	1
d660	Assisting others	1	1
d6600	Assisting others with self‐care	1	1
d7500	Informal relationships with friends	2	1
d7504	Informal relationships with peers	1	1
d770	Intimate relationships	1	1
d8502	Full‐time employment	1	1
d910	Community life	1	1
d920	Recreation and leisure	1	1
d9201	Sports	1	1
d9204	Hobbies	1	1
d9300	Organised religion	1	1
d950	Political life and citizenship	1	1
e115	Products and technology for personal use in daily living	1	1
e1150	General products and technology for personal use in daily living	1	1
e1151	Assistive products and technology for personal use in daily living	1	1
e1158	Products and technology for personal use in daily living, other specified—mouthwash	1	1
e1158	Products and technology for personal use in daily living, other specified—brush	1	1
e1158	Products and technology for personal use in daily living, other specified—snap fastener	1	1
e1158	Products and technology for personal use in daily living, other specified—Gold Filling	1	1
e1158	Products and technology for personal use in daily living, other specified—temporary prothesis	5	1
e250	Sound	1	1
e255	Vibration	1	1
e345	Strangers	1	1
e360	Other professionals	1	1
e465	Social norms, practices and ideologies	2	1
e5701	Social security systems	2	1
e5902	Labour and employment policies	2	1
s1201	Spinal nerves	1	1
s220	Structure of eyeball	1	1
s230	Structures around eye	1	1
s240	Structure of external ear	1	1
s32008	Other specified teeth—tooth enamel	1	1
s32020	Hard palate	1	1
s3204	Structure of lips	1	1
s7108	Structure of head and neck region, other specified_face	1	1
s810	Structure of areas of skin	1	1

### 3.4 Body Functions

The most frequently mentioned ICF Body Functions categories were b5101 Biting, b28010 Pain in head and neck, b152 Emotional functions, b1801 Body image, b28018 Pain in body part, other specified—tooth pain. The examples and text passages presented are not intended to be exhaustive but are intended to provide insights into the discussions and highlight significant concepts. Table 4 displays all identified ICF codes within the Body Functions domain, showing the frequency of mentions across all focus groups/interviews, as well as the number of focus groups/interviews in which each category was mentioned.

**TABLE 4:** ICF codes body functions.

ICF code	ICF concept	Frequency (absolute) across all focus groups/interviews	Number of focus groups and interviews in which the category was mentioned (*N* = 8)
b5101	Biting	16	8
b28010	Pain in head and neck	14	7
b152	Emotional functions (G)	20	6
b1801	Body image	32	5
b28018	Pain in body part, other specified_toothpain	11	4
b28018	Pain in body part, other specified_headache	7	4
b5102	Chewing	5	4
b1265	Optimism	7	3
b1266	Confidence	6	3
b265	Touch function	6	3
b280	Sensation of pain (G)	4	3
b28018	Pain in body part, other specified_mouthpain	4	3
b515	Digestive functions	3	3
b1300	Energy level	3	2
b140	Attention functions	2	2
b4350	Immune response	3	2
b4552	Fatigability	2	2
b5105	Swallowing	2	2
b530	Weight maintenance functions	3	2
b1260	Extraversion	1	1
b1301	Motivation	1	1
b134	Sleep functions	1	1
b1470	Psychomotor control	1	1
b1564	Tactile perception	1	1
b210	Seeing functions	1	1
b2153	Functions of lachrymal glands	1	1
b250	Taste function	2	1
b260	Proprioceptive function	1	1
b2700	Sensitivity to temperature	2	1
b2701	Sensitivity to vibration	1	1
b2702	Sensitivity to pressure	1	1
b28016	Pain in joints	1	1
b28018	Pain in body part, other specified_pain in neck	3	1
b28018	Pain in body part, other specified_pain in ears	1	1
b28018	Pain in body part, other specified_facepain	1	1
b320	Articulation functions	2	1
b435	Immunological system functions	1	1
b440	Respiration functions	1	1
b4500	Functions of breathing through the mouth	2	1
b5103	Manipulation of food in the mouth	1	1
b5104	Salivation	1	1
b5151	Breakdown of food	1	1
b5152	Absorption of nutrients	2	1
b520	Assimilation functions	1	1
b5350	Sensation of nausea	1	1
b5351	Feeling bloated	1	1
b7101	Mobility of several joints	1	1
b7300	Power of isolated muscles and muscle groups	1	1
b735	Muscle tone functions	1	1
b7651	Tremor	2	1
b7801	Sensation of muscle spasm	1	1

#### 3.4.1 b5101 Biting

…and when there were too many gaps, it was (difficult) to bite…

The ICF code b5101 Biting was identified 16 times across all focus groups and interviews. Most older participants reported difficulty biting prior to treatment, due to missing teeth. Others described being unable to bite properly because of tooth pain.

#### 3.4.2 b28010 Pain in Head and Neck

… you tense up and realize that it's starting to hurt…

The ICF code b28010 Pain in head and neck was identified 14 times in seven of the eight focus groups and interviews. Participants described radiating pain (see quote). Beyond tooth‐related pain, participants also reported pain in the temporomandibular joints and cramped jaw muscles.

#### 3.4.3 b152 Emotional Functions

… and doing this for years (puts his hand in front of his mouth to hide his bad teeth), that really put a strain on the psyche, that was mentally difficult

This ICF code b152 Emotional functions was identified 20 times in six of the eight focus groups and interviews. Many participants described experiencing negative emotions related to their oral health conditions (see quote above). While some reported fear of dental visits, other expressed joy in attending regular dental hygiene appointments.

#### 3.4.4 b1801 Body Image

When I stand in front of the mirror, I like it, I feel beautiful again

In the ICF code b1801 Body image was identified 32 times in five of the eight focus groups and interviews. Most participants reported a positive shift in self‐perception after dental restoration (see quote above). Before treatment, some shared that they avoided mirrors or disliked seeing themselves in photos.

#### 3.4.5 b28018 Tooth Pain

… I think that tooth pain is one of the worst things there is…

The ICF code b28018 pain in body part, other specified_tooth pain was identified 11 times in four of the eight focus groups and interviews. Participants either directly identified tooth pain (see quote above) or described radiating pain as a related issue.

### 3.5 Body Structures

The most frequently mentioned ICF Body Structures categories were s320 Structure of Mouth, s3200 Teeth, s710 Structure of head and neck region, s3201 Gums and s7101 Bones of face. Table 5 displays all identified ICF codes within the Body Structures domain, showing the frequency of mentions across all focus groups/interviews, as well as the number of focus groups/interviews in which each category was mentioned.

**TABLE 5:** ICF codes body structures.

ICF code	ICF concept	Frequency (absolute) across all focus groups/interviews	Number of focus groups and interviews in which the category was mentioned (*N* = 8)
s320	Structure of mouth	13	8
s3200	Teeth	46	8
s710	Structure of head and neck region	10	6
s3201	Gums	8	5
s7101	Bones of face	8	4
s32008	Other specified teeth—root of tooth	4	3
s3203	Tongue	4	3
s1201	Spinal nerves	1	1
s220	Structure of eyeball	1	1
s230	Structures around eye	1	1
s240	Structure of external ear	1	1
s32008	Other specified teeth—tooth enamel	1	1
s32020	Hard palate	1	1
s3204	Structure of lips	1	1
s7108	Structure of head and neck region, other specified_face	1	1
s810	Structure of areas of skin	1	1

#### 3.5.1 s320 Structure of Mouth

I always have something wrong with my mouth, I've always had problems since I was a child…

The ICF code s320 Structure of Mouth was identified 13 times across all focus groups and interviews. Most participants described issues or improvement regarding their mouths (see quote above). Some of the participants described in more detail what was wrong with their teeth or gums, which were linked to other ICF codes, i.e., s3200 Teeth or s3201 Gums.

#### 3.5.2 s3200 Teeth

… the teeth in the back are supported with metal, that they don't break again

The ICF code s3200 teeth was identified 46 times across all focus groups and interviews. Most participants described how the appearance of their teeth look (see quote above). Some participants described how the look of their teeth changed over time, or they described how they looked before and after a dental intervention.

#### 3.5.3 s710 Structure of Head and Neck Region

… I noticed that everything was cramped … Just everything that came from here (points to the jaw) and the neck.

The ICF code s710 Structure of head and neck region was identified 10 times in six of the eight focus groups and interviews. Most participants described tension or muscle stiffness (see quote above), while others described swelling caused by a dental infection.

#### 3.5.4 s3201 Gums

… the gums have somehow retracted…

The ICF code s3201 Gums was identified eight times in five of the eight focus groups and interviews. Most participants described that their gums had receded. Most older participants stated gum retraction mostly related to periodontitis (gum disease).

#### 3.5.5 s7101 Bones of Face

I will probably get the upper (implants), but maybe I will have to have a (bone) augmentation because the bone has receded

The ICF code s7101 Bones of face was identified 8 times in four of the eight focus groups and interviews. Some participants stated that they did not have enough bone, making implants more challenging (see quote above).

### 3.6 Activities and Participation

The most frequently mentioned ICF Activities and Participation categories were d5201 Caring for teeth, d5702 Maintaining one's health, d550 Eating, d870 Economic self‐sufficiency and d9205 Socialising. Table 6 displays all identified ICF codes within the Activities and Participation domain, showing the frequency of mentions across all focus groups/interviews, as well as the number of focus groups/interviews in which each category was mentioned.

**TABLE 6:** ICF codes activities and participation.

ICF code	ICF concept	Frequency (absolute) across all focus groups/interviews	Number of focus groups and interviews in which the category was mentioned (*N* = 8)
d5201	Caring for teeth	72	8
d5702	Maintaining one's health	43	8
d550	Eating	22	7
d870	Economic self‐sufficiency	27	6
d9205	Socialising	20	6
d240	Handling stress and other psychological demands	7	6
d5701	Managing diet and fitness	12	6
d740	Formal relationships	8	6
d349	Communication—producing, other specified and unspecified—laughing	11	5
d560	Drinking	4	4
d850	Remunerative employment (G)	7	4
d760	Family relationships	4	3
d7600	Parent–child relationships	3	3
d8451	Maintaining a job	6	3
d230	Carrying out daily routine (G)	4	2
d330	Speaking	2	2
d332	Singing	4	2
d7400	Relating with persons in authority	3	2
d7701	Spousal relationships	2	2
d175	Solving problems	1	1
d2301	Managing daily routine	1	1
d3150	Communicating with—receiving—body gestures	4	1
d350	Conversation	2	1
d440	Fine hand use	1	1
d470	Using transportation	2	1
d5208	Caring for body parts, other specified_putting on make‐up	1	1
d640	Doing housework	1	1
d660	Assisting others	1	1
d6600	Assisting others with self‐care	1	1
d7500	Informal relationships with friends	2	1
d7504	Informal relationships with peers	1	1
d770	Intimate relationships	1	1
d8502	Full‐time employment	1	1
d910	Community life	1	1
d920	Recreation and leisure	1	1
d9201	Sports	1	1
d9204	Hobbies	1	1
d9300	Organised religion	1	1
d950	Political life and citizenship	1	1

#### 3.6.1 d5201 Caring for Teeth

I really only learned how to brush my teeth properly at the ZMK (dental clinic). How to really brush your teeth. I didn't know that before

The ICF code d5201 Caring for teeth was identified 72 times across all focus groups and interviews. Most participants described their oral hygiene routine, describing not only how they brush their teeth, but also how they take care of their prostheses. Some of them pointed out, that they did not know how to properly take care of their teeth (see quote above). Some elderly or geriatric participants reported that food got stuck in their prostheses.

#### 3.6.2 d5702 Maintaining One's Health

… at most they file away some enamel, (so) I stick to the dentist's recommendation

The ICF code d5201 Caring for teeth was identified 43 times across all focus groups and interviews. Most participants mentioned that they follow a professional's recommendation, for example, the dentist's recommendation (see quote above). Some participants emphasised that they try to get annual check‐ups and regular cleanings at the dental office.

#### 3.6.3 d550 Eating

For a while I was only allowed to eat soft foods.

The ICF code d550 Eating was identified 22 times in seven of the eight focus groups and interviews. Participants commonly described difficulties related to eating, including challenges with specific foods, experience of teeth breaking during meals, and discomfort or issue eating in social settings.

#### 3.6.4 d870 Economic Self‐Sufficiency

I knew something had to happen. I was worried about my finances. I already knew that it would probably be a costly affair…

The ICF code d870 Economic self‐sufficiency was identified 27 times in six of the eight focus groups and interviews. Most participants were worried that they could not afford the dentist. Some of the participants stated that they knew that they would have to repair a lot and that it would cost a lot (see quote above). Elderly or geriatric participants emphasised that having some money on the side for dental treatments is important for them.

#### 3.6.5 d9205 Socialising

…social life depends extremely on it. If you feel good (about your teeth), you are integrated in social life.

The ICF code d9205 Socialising was identified 20 times in six of the eight focus groups and interviews. Participants reported challenges related to eating in a social setting, with some completely avoiding conversations, or, in one case, refraining from participating in social life altogether.

### 3.7 Environmental Factors

The most frequently mentioned Environmental Factors categories were e5800 Health Services, e310 Immediate family, e1158 Products and technology for personal use in daily living, other specified_implant, e355 Health professionals and e5801 Health Systems. Table 7 displays all identified ICF codes within the Environmental Factors domain, showing the frequency of mentions across all focus groups/interviews, as well as the number of focus groups/interviews in which each category was mentioned.

**TABLE 7:** ICF codes environmental factors.

ICF code	ICF concept	Frequency (absolute) across all focus groups/interviews	Number of focus groups and interviews in which the category was mentioned (*N* = 8)
e5800	Health services	88	8
e310	Immediate family	29	8
e355	Health professionals	45	7
e5801	Health systems	36	7
e1158	Products and technology for personal use in daily living, other specified—implant	18	7
e1100	Food	10	6
e1158	Products and technology for personal use in daily living, other specified—interdental brush	10	6
e1158	Products and technology for personal use in daily living, other specified—bridge	12	6
e325	Acquaintances, peers, colleagues, neighbours and community members	10	6
e1101	Drugs	19	5
e1158	Products and technology for personal use in daily living, other specified—prothesis	18	5
e1158	Products and technology for personal use in daily living, other specified—electric toothbrush	6	5
e1158	Products and technology for personal use in daily living, other specified—toothbrush	7	5
e1650	Financial assets	9	5
e1158	Products and technology for personal use in daily living, other specified—toothfloss	9	4
e320	Friends	7	4
e5700	Social security services	14	4
e1158	Products and technology for personal use in daily living, other specified—crown	3	3
e1158	Products and technology for personal use in daily living, other specified—toothpick	3	3
e315	Extended family	5	3
e1158	Products and technology for personal use in daily living, other specified—denture	2	2
e1158	Products and technology for personal use in daily living, other specified—braces	2	2
e1158	Products and technology for personal use in daily living, other specified—mouthguard	2	2
e1158	Products and technology for personal use in daily living, other specified—Amalgam Filling	5	2
e1158	Products and technology for personal use in daily living, other specified—post crown	2	2
e330	People in positions of authority	2	2
e350	Domesticated animals	2	2
e410	Individual attitudes of immediate family members	2	2
e460	Societal attitudes	2	2
e115	Products and technology for personal use in daily living	1	1
e1150	General products and technology for personal use in daily living	1	1
e1151	Assistive products and technology for personal use in daily living	1	1
e1158	Products and technology for personal use in daily living, other specified—mouthwash	1	1
e1158	Products and technology for personal use in daily living, other specified—brush	1	1
e1158	Products and technology for personal use in daily living, other specified—snap fastener	1	1
e1158	Products and technology for personal use in daily living, other specified—Gold Filling	1	1
e1158	Products and technology for personal use in daily living, other specified—temporary prothesis	5	1
e250	Sound	1	1
e255	Vibration	1	1
e345	Strangers	1	1
e360	Other professionals	1	1
e465	Social norms, practices and ideologies	2	1
e5701	Social security systems	2	1
e5902	Labour and employment policies	2	1

#### 3.7.1 e5800 Health Services

My treatment lasted almost a day each time, from 1‐5 pm, then I had to leave home at 11 am if you go by public transport

The ICF code e5800 Health Services was identified 88 times across all focus groups and interviews. Participants mentioned going to the dentist for regular check‐ups, to undergo complete dental restoration or to get a dental cleaning depending on their oral health state. Only a few participants mentioned getting emergency treatment at a hospital. Based on the personal experiences of the participants, this ICF code was described both as a facilitator and as a barrier (see quote above).

#### 3.7.2 e310 Immediate Family

Sometimes I almost didn't dare to open my mouth even within the family. I can remember my mother making comments from time to time and of course that influenced me. I was never proud of my teeth

The ICF code d5201 Caring for teeth was identified 29 times across all focus groups and interviews. Most participants described family members, such as siblings, children, and parents as facilitators, often providing motivation to seek dental treatment.

However, a few participants described family members as a barrier to their oral health (see quote above).

#### 3.7.3 e355 Health Professionals

I also had bad experiences with dentists before…

The ICF code e355 Health professionals was identified 45 times in seven of the eight focus groups and interviews. Participants mostly described going to the dentist or the dental hygienist, but a few participants mentioned medical doctors as well. Some participants mentioned health professionals, such as dentists, as a barrier (see quote above). A few of these participants mentioned that the dental profession has developed positively. When described as a barrier, some participants stated that they were overwhelmed by the different professional/expert opinions when seeking a second opinion on their oral health issue.

#### 3.7.4 e5801 Health Systems

It is of course completely unsatisfactory that one of the most important medical problems, namely dental problems, is not covered by general health insurance.

The ICF code e5801 Health Systems was identified 36 times in seven of the eight focus groups and interviews. Participants mostly mentioned dental clinics and hospitals, but others mentioned insurance as well. Both dental clinics and hospitals were equally described as facilitators as well as barriers depending on the participant's personal experiences. Participants mentioned the insurance as a barrier, mostly because certain treatments were not covered. A few participants mentioned insurance as a facilitator, since it partially or fully covered their dental treatment (see quote above).

#### 3.7.5 e1158 Products and Technology for Personal Use in Daily Living, Other Specified_Implant

… I had implants that were broken and were in at an angle…

The ICF code e1158 Products and technology for personal use in daily living, other specified_implant was identified 18 times in seven of the eight focus groups and interviews. Participants predominantly discussed implants in the context of dental restorations they had received. Some shared experiences of complications during or after the procedure, as highlighted in the accompanying quote. Others described the necessity of tooth extractions, which were followed by the placement of implants. Additionally, several participants noted challenges related to bone health, such as osteoporosis or insufficient bone structure, which prevented them from receiving implants.

### 3.8 Personal Factors

I have seen people who are 10 or 20 years older than me, who may not necessarily have a nice teeth positioning, but they still had their own teeth, fully organic, and I'm not retired yet and I already have dentures, and then I realized how jealous I am and I feel older. Maybe one day I get used to it, but at the moment I have to learn to let go…

(Personal Factor: Jealousy, feeling)

In total we identified 102 different personal factors, for example age, gender, coping strategies, personal characteristics, habits, etc. There are no ICF codes for personal factors.

### 3.9 Not Covered Codes

I can't remember, so I would have to look at the x‐ray.

In total we identified 34 codes that are not covered. For example, in the quote above x‐rays are not covered by the ICF and this concept was therefore marked as not covered.

### 3.10 Non‐Definable Codes

I do notice it. It's not something that bothers me right now.

We identified a total of 49 non‐definable codes, primarily because the concepts could not be linked to a specific ICF code. For example, in the quote above, the patient mentions something is “bothering” him, but the exact meaning remains unclear. It is uncertain whether the participant is referring to an uncomfortable sensation, mild pain, or dissatisfaction with appearance. Due to the lack of contextual clarity, the concept was marked as non‐definable.

### 3.11 Focus Group With Young Health Professionals

After linking the last focus group, consisting of young health professionals with oral health conditions, a lot of new information emerged, leading to the decision to display their results separately. In this focus groups a total of 309 meaningful concepts were extracted/coded and subsequently linked to a total of 78 ICF categories. The 77 categories comprise of 21 Body Functions, 24 Activities and Participation categories, 13 Body Structures, and 20 Environmental Factors. 15 categories were Personal Factors and 6 were non‐definable concepts. The degree of agreement was 64%. Table 8 presents the frequency of each ICF category mentioned within this focus group. Categories that were exclusively mentioned in this group, and not in any of the other eight focus groups or interviews, are marked in bold to highlight their unique contributions.

**TABLE 8:** ICF codes focus group comprising of young health professionals.

ICF code	ICF concept	Absolute frequency
b1265	Optimism	1
b1266	Confidence	4
b1300	Energy level	3
b134	Sleep functions	1
b140	Attention functions	1
b152	Emotional functions (G)	5
b1801	Body image	19
b265	Touch function	1
b2700	Sensitivity to temperature	1
**b2703**	**Sensitivity to a noxious stimulus**	7
b280	Sensation of pain (G)	1
b28010	Pain in head and neck	7
b28018	Pain in body part, other specified_pain in neck	1
b28018	Pain in body part, other specified_headache	1
b28018	Pain in body part, other specified_toothpain	3
b4350	Immune response	1
b5101	Biting	7
b5102	Chewing	1
b5103	Manipulation of food in the mouth	3
b7101	Mobility of several joints	1
**b740**	**Muscle endurance functions**	1
**d177**	**Making decisions**	1
**d210**	**Undertaking a single task**	1
**d220**	**Undertaking multiple tasks**	2
d240	Handling stress and other psychological demands	7
d330	Speaking	1
d349	Communication—producing, other specified and unspecified—laughing	1
**d4105**	**Bending**	2
**d4300**	**Lifting**	2
**d5101**	**Washing whole body**	1
d5201	Caring for teeth	10
d550	Eating	1
d560	Drinking	1
**d570**	**Looking after one's health**	3
d5701	Managing diet and fitness	10
d5702	Maintaining one's health	12
**d630**	**Preparing meals**	1
d660	Assisting others	1
d740	Formal relationships	2
**d750**	**Informal social relationships**	2
d7500	Informal relationships with friends	1
**d8450**	**Seeking employment**	2
d850	Remunerative employment (G)	2
d870	Economic self‐sufficiency	6
d9205	Socialising	2
e1100	Food	2
e1101	Drugs	3
e1151	Assistive products and technology for personal use in daily living	1
e1158	Products and technology for personal use in daily living, other specified—implant	2
e1158	Products and technology for personal use in daily living, other specified—crown	2
e1158	Products and technology for personal use in daily living, other specified—braces	10
e1158	Products and technology for personal use in daily living, other specified—mouthguard	1
**e1158**	**Products and technology for personal use in daily living, other specified—veneer**	1
**e1158**	**Products and technology for personal use in daily living, other specified**—**toothpaste**	2
e310	Immediate family	4
e325	Acquaintances, peers, colleagues, neighbours and community members	5
e345	Strangers	1
e355	Health professionals	7
e460	Societal attitudes	3
**e5500**	**Legal services**	1
e5700	Social security services	4
e5800	Health services	38
e5801	Health systems	14
**e585**	**Education and training services, systems and policies**	1
**e5851**	**Education and training systems**	1
s320	Structure of mouth	1
s3200	Teeth	12
**s32000**	**Primary Dentition**	1
s32008	Other specified teeth—tooth enamel	1
s32008	Other specified teeth—root of tooth	
s3201	Gums	1
**s3202**	**Structure of palate**	1
s3204	Structure of lips	1
**s3208**	**Structure of mouth, other specified**—**periodontium**	1
s710	Structure of head and neck region	5
s7101	Bones of face	9
**s7103**	**Joints of head and neck region**	2
**s7104**	**Muscles of head and neck region**	1

## 4 Discussion

We identified functioning‐based outcomes (ICF categories) for persons with an oral health condition in every component of the ICF. This indicates that oral health conditions affect different aspects of a person's everyday life. This also underscores the importance of considering functioning‐based outcomes when assessing patient needs and guiding intervention decision‐making.

### 4.1 Living With Oral Health Conditions

Oral health conditions have a biopsychosocial impact on an individual's life [7]. This was reflected in the statements made by the study participants. For most dental clinicians, it is clear that there is a direct relationship between oral health conditions and specific medical conditions [5]. Statements from some participants seem to support a link between their oral health conditions and other general health issues. Furthermore, many participants mentioned not only that they were unable to chew or bite, but also emphasised that they felt sad or frustrated that they couldn't eat certain foods anymore.

Emotions like sadness and frustration and other psychological/mental aspects related to dealing with an oral health condition were also mentioned in the context of lower self‐esteem, feeling stressed or not feeling good about themselves or orofacial appearance. With regard to psychological functioning, b152 Emotional Functions was one of the frequently identified codes in this study. This code refers to mental functions related to the regulation of emotions, which are essential for coping with stress and maintaining well‐being [9]. On a positive note, several participants agreed that being optimistic when dealing with an oral health condition is important. The psychological/mental issues related to the participants' oral health status, played an important role in their everyday life. To overcome or help with these issues, participants mentioned immediate family members, friends, or pets as major facilitators.

Friends or immediate family members were not always considered a facilitating environmental factor. Several participants mentioned that they felt ashamed of their teeth and had difficulties with socialising with family. Some participants even mentioned issues in romantic relationships and in finding a partner as well as getting a job due to potential barriers related to interactions with customers or co‐workers. On a positive note, many participants emphasised the importance of social support for living with an oral health condition and stated that they felt a sense of relief because others were struggling with the same issues. One focus group even exchanged phone numbers after the discussion to stay in contact [6, 9, 28].

### 4.2 Oral Health Assessment Tools

As previously indicated, this study is intended to inform the development of a set of functioning outcomes operationalised with ICF categories, the so‐called ICF Core Set for oral health. The ICF Core Set for oral health will serve as the basis for developing a tool for the assessment and reporting of functioning of persons with an oral health condition. This tool is not intended to replace existing tools but to complement them. For example, the Oral Health Impact Profile (OHIP) is one of the most commonly used oral health‐related Quality of Life instruments and consists of 49 questions aiming to capture functional restrictions, physical discomfort, psychological distress, physical and psychological impairment, and social impairment [29]. The main difference between the OHIP and the planned ICF Core Set is that the OHIP measures quality of life and the ICF measures functioning. While the term functioning captures the dynamic interaction between the functions and structures of the body with the ability to perform tasks and activities and impacting contextual factors, quality of life includes fulfilment, well‐being, and happiness in a variety of life domains [9, 29]. A higher quality of life can be a result of good functioning, but the two are not the same [30]. Another widely used tool is the AOHSS—a standardised set of questions and guidelines to assess oral health outcomes in adults focusing on caries and periodontological disorders. This tool was primarily developed for clinical use, meaning that clinicians may use it to co‐produce plans with patients, share decision‐making processes with them, and track the progress of oral health outcomes over time [31]. Functional assessments complement OHIP and AOHSS by providing a thorough and universal approach to evaluating an individual's overall well‐being and functioning in daily life [9, 29, 31]. Moreover, the ICF is a standardised referencing system making data about oral health conditions consistent and comparable worldwide [9].

### 4.3 Dental Patient‐Reported Outcomes

In clinical practice and research, there is an evolving shift from disease‐oriented outcomes to dental patient‐reported outcomes (dPROs) enabling improved person‐centred care [32, 33, 34]. Within dentistry, numerous measurement tools, referred to as dental patient‐reported outcome measures (dPROMs) are available to assess these dPROs [35, 36]. However, current dPROM's have limitations and there is a need for developing standardised protocols to improve data comparability [34, 35, 36]. Moreover, there is an increasing demand for incorporating PROs in the development of these standards [35]. This study emphasises the patient perspective and underscores the significance of identifying PROs. The results of the present study could contribute to promoting patient‐centred dental care by providing the basis for developing a patient‐informed, functioning‐based tool for the assessment and reporting of patients' oral health.

## 5 Limitations

This study has a few limitations in terms of bias. Selection bias may have been present, as recruitment predominately took place in three locations, all of which are located in the German‐speaking part of Switzerland. In the future, this could be addressed by including more study centers in other regions of Switzerland and internationally to better represent the oral health state of the general population worldwide. This would also enhance the transferability of the findings.

Regarding potential sampling bias, this was a convenience sample. The three recruitment centers were asked to invite potential participants who fulfilled the inclusion criteria. Participants who agreed to participate—after being approached by the dental team—may have been more aware of their oral health condition and more motivated to share their experiences than those who declined. Moreover, as one of the inclusion criteria was fluency in German, French or Italian speakers were excluded, which may also have influenced the findings.

In terms of credibility, we implemented strategies such as independent coding and consensus discussions to ensure consistency and transparency in the analysis. To enhance confirmability, both investigators independently linked all concepts using standardised ICF linking rules, with discrepancies resolved through discussion. The main investigator documented all coding and linking steps in detail. Regarding dependability, we followed established analytical procedures and maintained a detailed audit trail throughout the study. These measures contribute to the overall trustworthiness of the findings.

## 6 Conclusion

This study identified 150 ICF categories that represent the most important and relevant functioning‐based outcomes for persons with oral health conditions, as expressed by participants during focus groups and interviews. These outcomes span all components of the ICF, underscoring the broad impact of oral health conditions on daily life. The findings provide a patient‐centered foundation for developing standardised tools and reporting systems in oral health care. Specifically, they will inform the selection of categories during the upcoming consensus conference to develop the ICF Core Set for oral health [16]. Future research will focus on developing and pilot‐testing a practical assessment tool based on this Core Set [12].

## 7 AI‐Based Tools

- DeepL Translate: https://www.deepl.com/de/translator
  ○ Translation of text passages
- Grammarly: https://app.grammarly.com/
  ○ Grammar and spelling review
- ResearchRabbit: https://www.researchrabbit.ai/
  ○ Visual overview over literature

## Author Contributions

**C. Lenherr:** (Main study investigator) conceptualization of study; preparation of ethics proposal; coordinated and conducted patient recruitment; Planned coordinated and conducted, all focus groups and interviews; transcribed and analysed (coded and linked) the data from all focus groups and interviews. **M. Schimmel:** provided institutional support including providing contacts to dental practices in the Bern region and financial support for recruitment activities; provided input for the study conceptualization; reviewed the ethics proposal. **G. Stucki:** provided input for the study conceptualization; provided contacts to dental practices in the Luzern region. **M. Selb:** (Second investigator) provided input for the study conceptualization; supported the development and finalisation of the ethics proposal; supported the preparation for conducting the focus groups/interviews; served as the second investigator during the coding and linking process.

## Conflicts of Interest

Prof. Martin Schimmel is an associate editor of the Journal of Oral Rehabilitation. To avoid any potential conflicts of interest, they were not involved in the editorial review or decision‐making process for this manuscript. The remaining authors declare no conflicts of interest.

## Supporting information


**Data S1:** joor70078‐sup‐0001‐DataS1.pdf.

## Data Availability

The data that support the findings of this study are available from the corresponding author on reasonable request.
