# Supplementary material for: Identification of Important and Relevant Functioning‐Based Outcomes for Persons With an Oral Health Condition From the Patient's Perspective
Source: J Oral Rehabil. 2025 Oct 15;53(1):211–35. doi: 10.1111/joor.70078 (PMC12705294; doi:10.1111/joor.70078)
Supplement: Supplementary file 1 — Data S1: joor70078‐sup‐0001‐DataS1.pdf. [file JOOR-53-211-s001.pdf]

UNIVERSITÄT
LUZERN

b UNIVERSITÄT BERN

Questionnaire

Please fill out in block letters

Participant Number:	
Age (in years):	
Gender	☐ Male<br>☐ Female<br>☐ other
Martial Status	☐ Never married<br>☐ Married<br>☐ In a registered partnership<br>☐ Seperated<br>☐ Divorced<br>☐ Widowed<br>☐ other: ____________
Living Situation (e.g. nursing home)	☐ Alone<br>☐ With another person (e.g. partner, nursing home)
Employment Status	☐ Employed<br>☐ Self-employed<br>☐ Unpaid work (e.g. volunteer work)<br>☐ In education / student<br>☐ Housewife/househusband<br>☐ Retired<br>☐ Unemployed<br>☐ Unable to work. Please also answer next question.<br>☐ other: ____________
If permanently unable to work or absent (due to education/studies), please check the reason	☐ Oral cancer<br>☐ Jaw necrosis<br>☐ Due to another problem with the mouth, teeth, or dentures<br>☐ Other reason:
Highest Level of Education	☐ No formal education<br>☐ Primary education<br>☐ Secondary education<br>☐ Apprenticeship<br>☐ Swiss University entrance qualification (Matura)<br>☐ University of Applied Sciences/University (Bachelor's, Master's)<br>☐ Doctorate/PhD<br>☐ other: ____________
Income Level (per month in CHF)	☐ Up to 5000<br>☐ 5'000 – 10'000<br>☐ 10'000 – 15'000<br>☐ 15'000 – 20'000<br>☐ More than 20'000

Do you have problems with?	☐ Sense of taste<br>☐ Tactile sensation in oral cavity<br>☐ Breathing<br>☐ Yawning<br>☐ Biting<br>☐ Chewing<br>☐ Salivation<br>☐ Swallowing	☐ Facial expression an mimicry<br>☐ Aesthetic of the face<br>☐ Dry mouth<br>☐ Kissing<br>☐ Speaking<br>☐ Singing<br>☐ Eating with others
How long have you had any pain in your jaw or temple area on one or both sides in the past 30 days?	☐ No pain<br>☐ Pain comes and goes<br>☐ Pain is constant	
Personal Oral Hygiene<br>(e.g., brushing teeth, denture care, flossing/interdental brushes)	☐ Three times a day or more<br>☐ Usually twice a day<br>☐ Usually once a day<br>☐ Several times a week<br>☐ Less than once a week<br>☐ Almost never	
Do you have any of the following dental prostheses? (multiple answers possible)	☐ Removable prosthesis (e.g., full denture, partial denture)<br>☐ Fixed prosthesis (e.g., implant crown, bridges)	
Smoking Status	☐ Non-smoker<br>☐ Former smoker<br>☐ Smoker	
General Health Conditions<br>(multiple answers possible)	☐ No general diseases<br>☐ Cancer<br>☐ Cardiovascular disease<br>☐ Chronic respiratory disease<br>☐ Diabetes<br>☐ Back pain<br>☐ Arthrosis (joint wear and tear)<br>☐ Arthritis (joint inflammation)<br>☐ Rheumatism<br>☐ Osteoporosis (bone loss)<br>☐ Stress or Depression<br>☐ Other: ____________________	
